# Optimization of allogeneic hematopoietic cell transplantation for patients with myelofibrosis treated with ruxolitinib: eligibility, best practices, and improving transplant outcomes

**DOI:** 10.1007/s00277-025-06270-9

**Published:** 2025-03-22

**Authors:** Sarah A. Wall, Roni Tamari, Zachariah DeFilipp, Gabriela S. Hobbs

**Affiliations:** 1 Division of Hematology, The Ohio State University, 2121 Kenny Road, James Outpatient Care, Office 7226, Columbus, OH 43210 USA; 2 Department of Medicine, Memorial Sloan Kettering Cancer Center, New York, NY USA; 3 Hematopoietic Cell Transplant and Cell Therapy Program, Massachusetts General Hospital, Boston, MA USA; 4 Division of Hematology/Oncology, Massachusetts General Hospital, Boston, MA USA

**Keywords:** Myelofibrosis, Ruxolitinib, Janus kinase, Myeloproliferative neoplasm

## Abstract

Allogeneic hematopoietic cell transplantation (HCT) is the only curative treatment for myelofibrosis (MF), and current guidelines recommend assessing all patients with MF for eligibility. Several patient- and disease-specific factors impact transplantation outcomes, and timely assessment of potential transplant candidates is key to optimizing post-HCT outcomes. The role of HCT in the treatment of MF continues to evolve, with the adoption of newer and safer approaches, enhanced donor availability, use of reduced-intensity conditioning, improvements in graft-versus-host disease (GVHD) prophylaxis and treatment, and greater understanding of high-risk clinical and molecular features of the disease. These developments highlight the importance of early and ongoing assessment throughout the MF disease course to optimize eligibility and consideration for HCT. Ruxolitinib is approved for first-line treatment of intermediate- or high-risk MF, and emerging data have clarified the important role of ruxolitinib in not only optimizing clinical status before HCT but also mitigating and treating post-HCT complications in patients with MF, notably acute and chronic GVHD and relapse. Here we review strategies for optimizing clinical outcomes in patients considered for and undergoing HCT for MF treated with ruxolitinib. We discuss strategies for appropriate patient and donor selection, optimization of ruxolitinib therapy in the pre- and peri-HCT periods, choice of conditioning regimen, GVHD prophylaxis, post-HCT management of GVHD, continued monitoring for MF relapse, and the role of post-HCT ruxolitinib maintenance to reduce risks of GVHD and disease relapse.

## Introduction

Myelofibrosis (MF) is a chronic, progressive myeloproliferative neoplasm (MPN) characterized by reticulin and/or collagen fibrosis in bone marrow with megakaryocytic proliferation and atypia [1]. Estimated MF prevalence ranges from 4 to 6 per 100,000 individuals in the United States [2] and from 2 to 4 per 100,000 worldwide [3]. Somatic driver mutations in the *JAK2*, *CALR*, or *MPL* genes occur in most patients. These mutations perpetuate cytokine signaling via the Janus kinase/signal transducer and activator of transcription (JAK/STAT) signaling pathway, which promotes cell proliferation and survival and activation of several inflammatory pathways [4–7]. Patients with MF have shorter overall survival (OS) compared with the other MPNs, such as essential thrombocythemia and polycythemia vera [8, 9], and the presence of additional somatic mutations and complex karyotype further impacts disease risk and OS [10]. In addition, constitutional symptoms are common, including fatigue, abdominal pain and early satiety due to splenomegaly, night sweats, bone pain, and pruritus [11], which can negatively impact patients’ quality of life.

JAK inhibitors (JAKi’s) remain the standard of care as primary treatment in most patients with newly diagnosed MF [12]. In 2024, there are 4 JAKi’s approved for the management of MF: ruxolitinib, approved for intermediate- or high-risk MF; fedratinib, approved for intermediate-2 or high-risk MF [13]; pacritinib, approved for intermediate- or high-risk MF with platelet count < 50 × 10^9^/L [14]; and momelotinib, approved for intermediate- or high-risk MF and anemia [15]. These agents have been shown to improve MF-related splenomegaly and symptoms. However, only ruxolitinib has the most robust data supporting an OS benefit, and no study has directly compared the other agents to ruxolitinib [16, 17]. Additionally, each JAKi has different specificity and potency, which explains the variability in clinical efficacy and unique toxicity profiles of these agents [18].

**Fig. 1:**
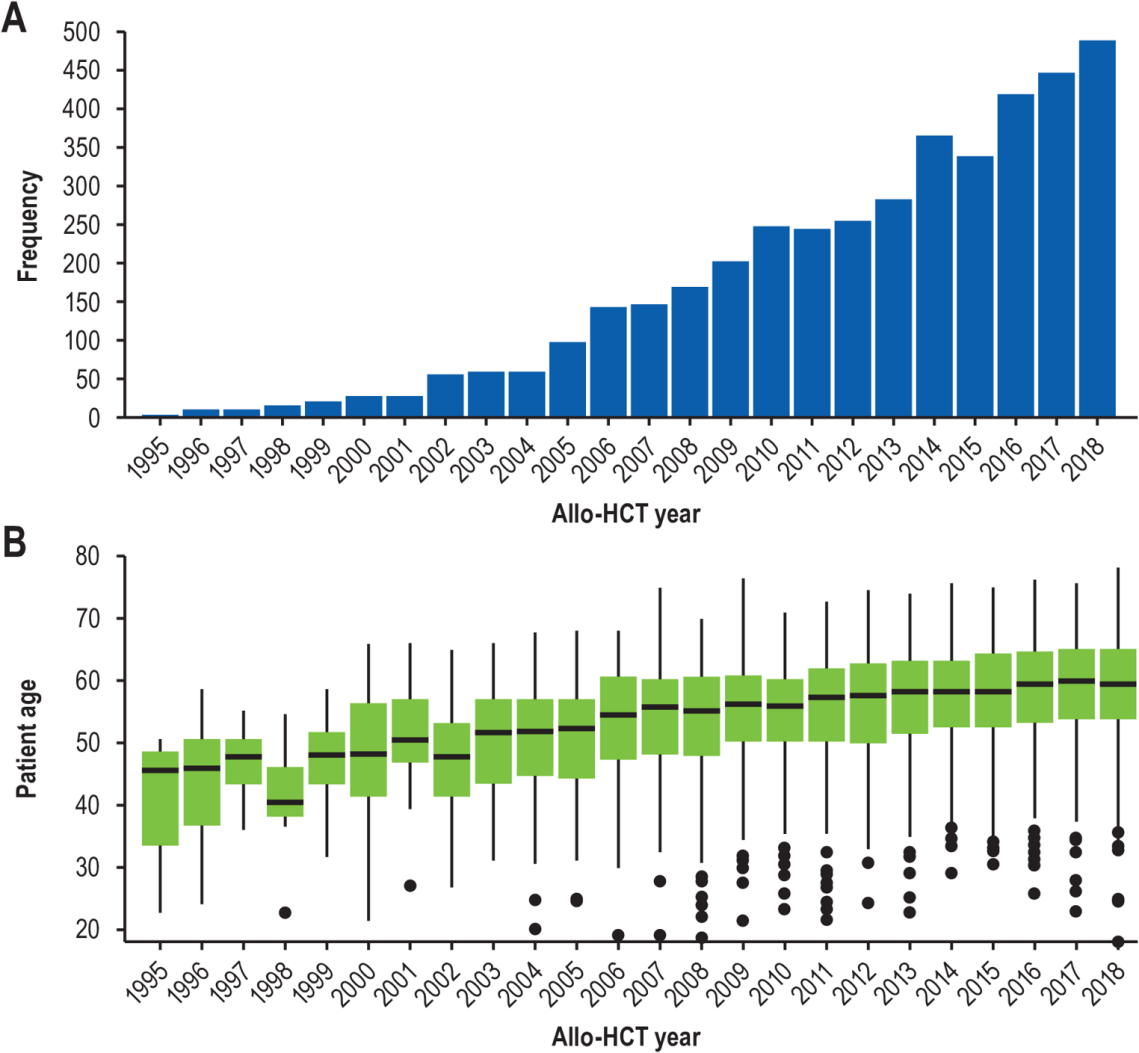
Trends in HCT for MF from 1995–2018. (**A**) Number of HCTs by year. (**B**) Changes in recipient age over time. allo-HCT; allogeneic HCT; HCT, hematopoietic cell transplantation; MF, myelofibrosis. This figure was reprinted with permission from Springer Nature. Sourced from McLornan D, et al. Trends in allogeneic haematopoietic cell transplantation for myelofibrosis in Europe between 1995 and 2018: a CMWP of EBMT retrospective analysis. *Bone Marrow Transplant*. 2021 Sep;56(9):2160–2172. 10.1038/s41409-021-01305-x

Allogeneic hematopoietic cell transplantation (HCT) remains the only potentially curative treatment option for MF [19]. Although careful selection of patients to assess candidacy for HCT remains essential, advances in donor availability, graft-versus-host disease (GVHD) prophylaxis and treatment, and post-HCT care have improved transplantation outcomes [20–23], which has resulted in a continuous increase in the number of HCTs for MF over the last 2 decades (Fig. 1A) [24]. Furthermore, the adoption of reduced-intensity conditioning (RIC) has allowed for increased access to HCT that has lower toxicity than myeloablative conditioning (MAC) without compromising anti-leukemic activity. As a result, the median age at HCT has increased substantially from 49.4 years between 1995 and 2006 to 59.3 years from 2015 to 2018 (Fig. 1B) [25]. Unfortunately, only a small minority of patients who require HCT ultimately receive this therapy, either due to an underlying comorbid condition or because they do not receive a referral in time [26].

Emerging data indicate that ruxolitinib use in the pre- and peri-HCT periods can reduce spleen volume—a key correlative of post-HCT outcomes—and improve symptom burden, which may help optimize patient clinical status prior to undergoing HCT [27]. Recent studies suggest that ruxolitinib use in the post-HCT setting as part of a maintenance strategy can reduce the risk of acute and chronic GVHD and protect against disease relapse [28, 29]. This review describes these evolving transplantation practices and provides commentary on optimal management of patients undergoing HCT with MF.

## Clinical case– diagnosis and risk stratification

A 64-year-old man with *CALR-* and *ASXL1-*mutated MF initially presented for evaluation with night sweats, abdominal discomfort, and early satiety. A bone marrow biopsy showed grade 2 + fibrosis, no increased blasts, and a normal male karyotype. His white blood cell count was 8.0 × 10^3^/µL, hemoglobin was 9 g/dL, platelets were 87 × 10^9^/L, and he had 3% circulating blasts. The patient’s spleen was palpated 10 cm below the costal margin.

The patient was risk stratified and considered high risk by the Dynamic International Prognostic Scoring System (DIPSS) and the Mutation-Enhanced International Prognostic Scoring System (MIPSS) due to anemia, thrombocytopenia, *ASXL1* mutation, constitutional symptoms, and levels of circulating blasts.

### Patient eligibility and selection for HCT

In our practices, we evaluate all patients with MF for HCT eligibility, in line with current guidelines from the American Society for Transplantation and Cellular Therapy and the National Comprehensive Cancer Network [20, 21, 30]. The decision to move forward with HCT is based on several factors including patient preference, prognostic scoring system outcomes, comorbidity assessment (e.g., with the HCT Comorbidity Index score [31]), Karnofsky performance status, psychosocial status (e.g., mental health status, social status, functional capacity of the patient), and availability of a caregiver.

Prognostic scoring systems (Table 1) are available to guide treatment selection; however, in our experience, the best use of these metrics is as a companion to clinical judgment to help assess where the patient is in the general trajectory of the disease. These assessment tools should be used early in the patient relationship to ensure optimal time for HCT referral. In cases where patient characteristics change over time, many of these tools can be used over time to reassess their disease changes.

**Table 1:** Prognostic models in myelofibrosis

Parameters (points)				Score				
	DIPSS [32]	DIPSS+ [133]	MIPSS-70 [33]	MIPSS-70 + v2.0 [10]	GIPSS [134]	MYSEC-PM [135]	RR6 [71, 136]	MTSS [41]
Age, y	> 65 (1)	*DIPSS risk* • Low (0) • Int-1 (1) • Int-2 (2) • High (3)	–	–	–	Years × 0.15	–	≥ 57 (1)
Leukocytes, ×10^9^/L	> 25 (1)		> 25 (2)	–	–	–	–	> 25 (1)
Circulating blasts, %	≥ 1 (1)		≥ 2 (1)	≥ 2 (1)	–	≥ 3 (2)	–	–
Hemoglobin, g/dL	< 10 (2)		< 10 (1)	Moderate anemia (1); severe (2)^a^	–	< 11 (2)	–	–
Platelets, ×10^9^/L	–	< 100 (1)	< 100 (2)	–	–	< 150 (1)	–	< 150 (1)
JAK inhibitor	–	–	–	–	–	Ruxolitinib	Ruxolitinib	Ruxolitinib
Constitutional symptoms	Yes (1)	Yes (1)	Yes (1)	Yes (2)	–	Yes (1)	–	–
TD-anemia	–	Yes (1)	–	–	–	–	At 3 and/or 6 mo (1); at all time points (1.5)	–
BM fibrosis grade	–	–	≥ 2 (1)	–	–	–	–	–
Cytogenetics	–	Unfavorable^b^ (1)	–	Unfavorable (3); very high risk (4)^c^	Unfavorable (1); very high risk (2)^c^	–	–	–
Driver mutations	–	–	Non-*CALR* type 1 (1)	Non-*CALR* type 1 (2)	Non-*CALR* type 1 (1)	Non-*CALR* (2)	–	Non-*CALR* / *MPL* (2)
Other mutations	–	–	HMR present (1)^d^; ≥2 HMR mutations (2)	HMR present (2)^e^; ≥2 HMR mutations (3)	*ASXL1* (1), *SRSF2* (1), *U2AF1Q* (1)	–	–	*ASXL1* (1)
MTSS-specific parameters	–	–	–	–	–	–	–	–
Karnofsky status, %	–	–	–	–	–	–	–	< 90 (1)
Donor relation	–	–	–	–	–	–	–	Mismatched unrelated (2)
RR6-specific parameters	–	–	–	–	–	–	–	–
RUX dose	–	–	–	–	–	–	< 20 mg bid at all time points (1)	–
Spleen size	–	–	–	–	–	–	Palpable length reduction ≤ 30% of BL at months 3 and 6 (1.5)	–
Score range (median OS)	• Low: 0 (NR) • Int-1: 1–2 (14 y) • Int-2: 3–4 (4 y) • High: ≥5 (2 y)	• Low: 0 (15 y) • Int-1: 1 (7 y) • Int-2: 2–3 (3 y) • High: 4–6 (1 y)	• Low: 0–1 (NR) • Int: 2–4 (6 y) • High: ≥5 (3 y)	• Very low: 0 (NR) • Low: 1–2 (16 y) • Int: 3–4 (8 y) • High: 5–8 (4 y) • Very high: ≥9 (2 y)	• Low: 0 (26 y) • Int-1: 1 (8 y) • Int-2: 2 (4 y) • High: ≥3 (2 y)	• Low: <11 (NR) • Int-1: 11–13 (9 y) • Int-2: 14–15 (4 y) • High: ≥16 (2 y)	• Low: 0 (NR) • Int: 1–2 (5 y) • High: ≥2.5 (3 y)	• Low: 0–2 (NR) • Int: 3–4 (14 y) • High: 5 (5 y) • Very high: 6–9 (1 y)

Numbers in parentheses indicate how many points are associated with each parameter and contributed to the total score. bid, twice daily; BL, baseline; BM, bone marrow; DIPSS, Dynamic International Prognostic Scoring System; GIPSS, Genetically Inspired Prognostic Scoring System; HMR, high molecular risk; Int, intermediate; JAK, Janus kinase; MIPSS-70, Mutation-Enhanced International Prognostic Scoring System; MTSS, Myelofibrosis Transplant Scoring System; MYSEC-PM, Myelofibrosis Secondary to Polycythemia Vera and Essential Thrombocythemia Prognostic Model; NR, not reached; OS, overall survival; RR6, response to ruxolitinib after 6 months; RUX, ruxolitinib; TD, transfusion dependent

^a^ Severe anemia: <8 g/dL in women, < 9 g/dL in men; moderate anemia: 8–9.9 g/dL in women, 9–10.9 g/dL in men

^b^ Complex karyotype or sole or 2 abnormalities including trisomy 8, − 7/7q–, i(17q), − 5/5q–, 12p–, inv(3), or 11q23 rearrangement

^c^ Unfavorable: chromosomal abnormalities except very high risk or sole 13q–, + 9, 20q–, chromosome 1 translocation/duplication or sex chromosome alterations including–Y; very high risk: single/multiple abnormalities of − 7, i(17q), inv(3)/3q21, 12p–/12p11.2, 11q–/11q23, + 21, or other autosomal trisomies except + 8/+9

^d^ Presence and number of *ASXL1*, *EZH2*, *SRSF2*, or *IDH1/2*

^e^ Presence and number of *ASXL1*, *EZH2*, *SRSF2*, *IDH1/2*, or *U2AF1Q157*

In our practices, we generally begin by using the DIPSS [32] as a preliminary assessment and the MIPSS [33, 34] once molecular data are available, often simultaneously. Unlike for acute leukemia for which HCT is pursued immediately after remission is achieved and a donor is secured, an area of unmet need in MF is determining HCT timing for even the highest-risk patients. The current risk scores in MF do not guide the clinician in the exact timing of HCT and simply provide an estimate of survival at different time points generally measured in years. Risk stratification scores are useful to determine if HCT is indicated but should not be used in isolation. For example, some patients with high-risk disease features may have relatively stable clinical disease, making it challenging to determine the optimal time for transplantation. This is particularly difficult for patients who experience significant improvement in spleen size and symptoms with JAKi’s. In addition, some patients have features that make transplant more urgent, such as transfusion dependence, which is among the most important indicators for proceeding with HCT quickly, as iron overload negatively impacts HCT outcomes and is associated with higher nonrelapse mortality [35].

Confirmed mutations in *ASXL1* and *TP53* also lower the threshold for proceeding to HCT, as they are associated with shortened OS, although the *ASXL1* effect may be limited to primary MF [36, 37]. However, there is conflicting evidence regarding the prognostic value of *ASXL1* and *TP53* mutations after HCT. There is evidence supporting a lack of correlation between these mutations and clinical outcomes (e.g., OS, relapse-free survival) [38], and other studies have suggested an association with unfavorable outcomes [39, 40]. Mutations in *ASXL1* have been shown to increase risk of relapse [39], and mutations in *TP53* increased the risks of relapse and leukemia transformation and reduced OS after HCT, particularly for multihit *TP53* mutations [40]. As with all HCT patients, we recommend monitoring for signs of relapse and progression (discussed below in the *Post-HCT optimization* section).

Beyond the standard MF prognostic scoring tools, the MF Transplant Scoring System (MTSS) is recommended by the European Society for Blood and Marrow Transplantation (EBMT)/European LeukemiaNet (ELN) to predict which patients may experience poor outcomes following HCT [41]. However, due to the lack of reproducibility with other data sets [41, 42], the MTSS is not used in isolation. There is also increasing use of artificial intelligence and machine learning in transplantation research [43]. Recent data suggest that machine learning may improve MF risk stratification with substantial predictive accuracy, leading to better treatment decisions and improved survival [44, 45]. Although we have not integrated machine learning tools into our clinical practice yet, we expect these will become increasingly relevant in coming years.

## Clinical case– initial management and HCT planning

The patient was started on ruxolitinib 10 mg twice daily (bid). After 2 months, his night sweats resolved, but his spleen remained enlarged (palpable 8 cm below the costal margin), and his hemoglobin dropped to 7.5 g/dL. At this point based on his high-risk features, the patient was evaluated for HCT. As part of HCT planning, the patient was also referred to radiation oncology for consideration of splenic irradiation.

### Pre-HCT patient management optimization

#### Initial treatment of MF

**Fig. 2:**
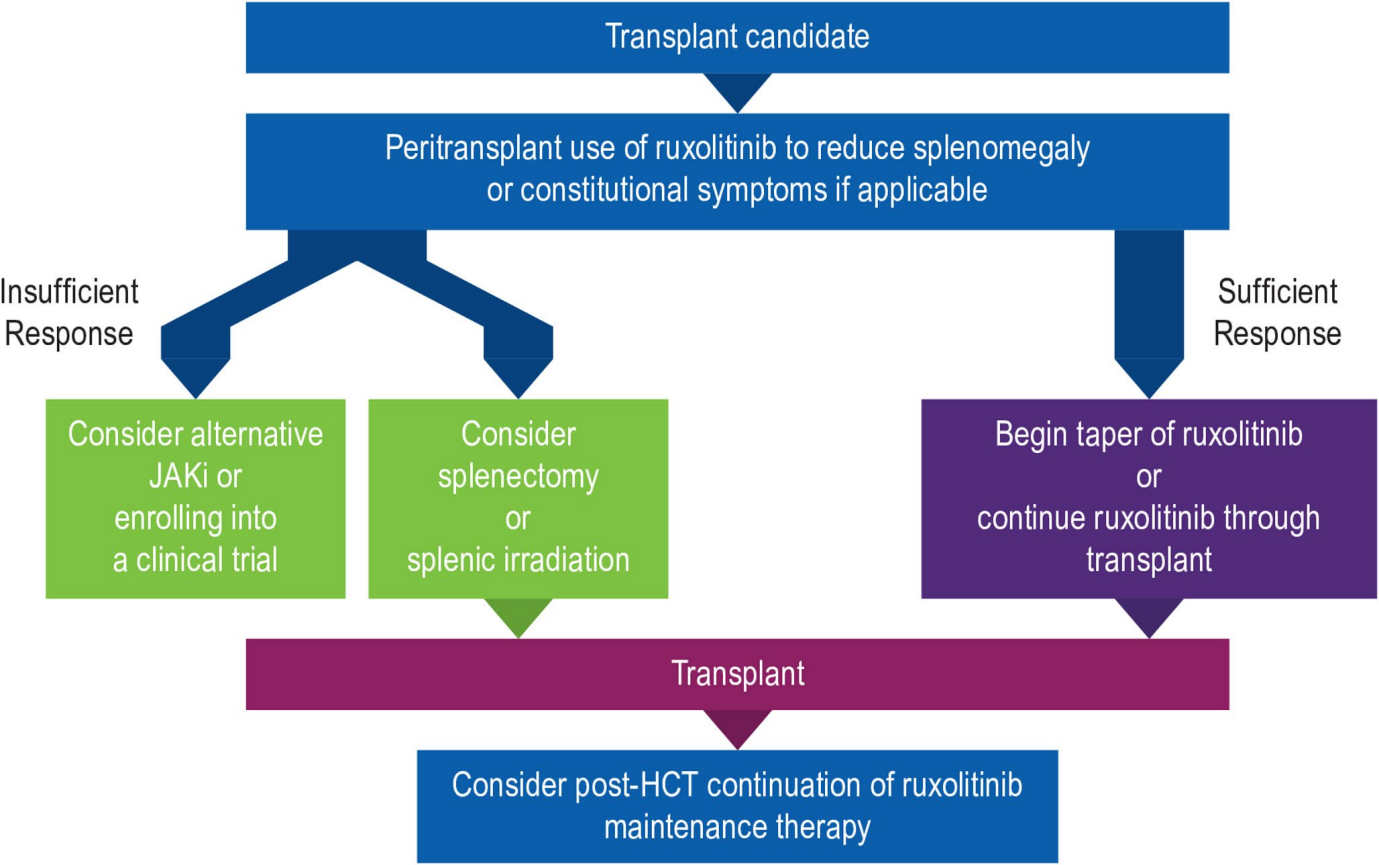
Proposed treatment algorithm during the pre-, peri-, and post-HCT Period. HCT, hematopoietic cell transplant; JAKi, janus kinase inhibitor

As part of a pre-HCT optimization strategy, we start patients on ruxolitinib (outside of compelling indications for alternative JAKi’s) as first-line option to reduce spleen size and improve symptom burden as recommended by the EBMT/ELN (Fig. 2) [46, 47]. Ruxolitinib optimization is important prior to HCT to achieve spleen and symptom control [48–51]. Improvements in splenomegaly are important, as larger spleen size is associated with slower engraftment and higher risk of relapse [52]. In addition, pre-HCT treatment with JAKi’s generally can improve patients’ functional status by ameliorating other MF-related symptoms, including fatigue, itching, night sweats, and bone/muscle pain [48, 53]. Pre-HCT ruxolitinib use has been evaluated in several real-world analyses, which indicate it is associated with low rates of graft failure, GVHD, and nonrelapse mortality, and some studies indicate that patients who have a spleen response with ruxolitinib prior to HCT have better HCT outcomes (Table 2) [50, 51, 54–63].

**Table 2:** Key studies describing ruxolitinib utilization in the pre- or peri-HCT period

Author, year	Patients, *N*	Study design	JAK use (pre/peri)	JAK inhibitor^a^ tapering strategy	Spleen response, %	Graft failure, %	Grade 2–4 aGVHD, %	NRM, % (landmark)	OS, % (landmark)
Jaekel, 2014 [50]	14	R	Pre	Taper and stop at conditioning	82^b^	7	29	7 (median f/u, 9 mo)	79 (median f/u, 9 mo)
Stübig, 2014 [51]	22	R	Pre	Stop at conditioning	45 (> 50% reduction); 24 (< 50%)	0	36	14 (1 y)	81 (1 y)
Shanavas, 2016 [60]	100	R	Pre	Variable^c^	23 (≥ 50% reduction)	8	37	28 (2 y)	61 (2 y)
Hanif, 2016 [61]	10	R	Pre	Taper started 6 d before conditioning	56 (≥ 10% reduction)	0	0 grade 3/4	0 (median f/u, 14.5 mo)	100 (median f/u, 14.5 mo)
Kröger, 2018 [62]	12	R	Peri	Taper started at + 20 d; stop at + 28 d post-HCT	100 (any improvement)	0	8	0 (median f/u, 17 mo)	100 (median f/u, 17 mo)
Shahnaz Syed Abd Kadir, 2018 [56]	46	R	Pre	Variable^d^	30 (> 50% reduction)	4	37	23 (2 y)	83 (2 y)
Pu, 2019 [63]	9	R	Pre	Taper started 5 d before conditioning	Median reduction, 11% (range, 16–40%)	0	0 grade 3/4	0 (36.5 mo)	100 (36.5 mo)
	4		Peri	Median 20 mo post-HCT	Median reduction, 32% (range, 20–38%)	0	0		
Gupta, 2019 [55]	21	P	Pre	Taper start 4 d before conditioning	36 (> 50% reduction)	16	43	28 (2 y)	66 (2 y)
Chhabra, 2020 [54]	37 (*n* = 32 received RUX)	R	Pre	Stopped median 7 d before conditioning	NA	8 (among RUX pts)	30	16 (2 y)	81 (3 y)
Salit, 2020 [59]	28	P	Pre	Taper during conditioning, stopped by 4 d before infusion	NA	0	78	7 (median f/u, 13 mo)	86 (2 y)
Ali, 2022 [67]	18	P	Peri	RUX tx − 3 d to + 30 d post-HCT	NA	0	17	23 (1 y)	77 (1 y)
Kröger, 2021 [57]	277	R	Pre	Tapered in 48%; discontinued before allograft for reason other than HCT in 23%	17 (> 50% reduction)	NA	28 (nonresponders^e^); 27 (responders^e^)	26 (nonresponders^e^), 15 (responders^e^) at 1 y	58 (nonresponders^e^), 70 (responders^e^) at 2 y
Robin, 2021 [58]	59	P	Pre	Tapered in 29%; abrupt discontinuation in 71%	20 (≥ 50% decrease if baseline ≤ 10 cm from LCM or ≥ 30% decrease if baseline ≥ 10 cm from LCM)	2	66	42 (1 y)	RUX tapered, 35 (2 y); Abrupt discontinuation, 59% (2 y)

aGVHD, acute graft-versus-host disease; f/u, follow-up; HCT, hematopoietic cell transplantation; JAK, Janus kinase; LCM, left costal margin; NA, not available; NRM, nonrelapse mortality; OS, overall survival; P, prospective; peri, peri-HCT; pre, pre-HCT; pts, patients; R, retrospective; RUX, ruxolitinib; tx, treatment

^a^ JAK inhibitor used in study was ruxolitinib unless otherwise noted

^b^ “Median reduction in palpable spleen below the costal margin of 41% in 9 of 11 patients with splenomegaly before ruxolitinib”

^c^ JAK inhibitors included ruxolitinib (*n* = 90 patients), momelotinib (*n* = 6), and other (*n* = 4); continued until stopped for HCT (*n* = 66), discontinued ≥ 1 mo before HCT (*n* = 30), not available (*n* = 4)

^d^*n* = 35 continued ruxolitinib until conditioning initiation, *n* = 11 discontinued ruxolitinib before conditioning because of no response or loss of response

^e^ Responder, ≥ 25% reduction in spleen size; nonresponder, < 25% reduction in spleen size

Based on preliminary results from a prospective phase 2 study [64], we consider continuing ruxolitinib use during the peri-HCT period (see following section [*Ruxolitinib strategy before HCT*]) [62, 64–67] in patients who have had a spleen response (spleen size reduction < 5 cm [68]) who will proceed to HCT. This strategy avoids the need for tapering therapy, which is done to mitigate risk of discontinuation syndrome, and diminishes potential for recrudesce of spleen enlargement and MF-associated symptoms during the peri-HCT period. Additionally, continuation of ruxolitinib during HCT does not appear to impact engraftment kinetics and is associated with low rates of both acute and chronic GVHD [64]. In our practices, we recommend tapering ruxolitinib to 5 mg bid during the peri-HCT period, with potential dose optimization upon engraftment post-HCT as tolerated. Now that additional JAKi’s are available for MF, we expect that there will be interest in evaluating safety and efficacy of these agents in the peri-HCT period, but currently, limited data are available. Preliminary findings from the phase 2 HOVON-134 trial involving 38 patients who underwent HCT suggest that pacritinib may be a safe and effective pre-HCT approach. The study results showed a low incidence of posttransplant failure (based on primary and secondary graft failure, grade III–IV acute GVHD, and death at 6 months) [69]; however, these findings need to be confirmed in a larger population.

#### Ruxolitinib strategy before HCT

Patients preparing for HCT who have MF-related symptoms or splenomegaly are started on a JAKi typically for at least 12 weeks before HCT to maximize spleen volume improvement [70], as splenomegaly reduction within 3 to 6 months of ruxolitinib treatment is associated with improved survival [71]. Ruxolitinib is usually started in patients with requisite blood counts, but alternative JAKi therapy with pacritinib or momelotinib may be considered in patients with severe thrombocytopenia (platelets < 50 × 10^9^/L) or anemia, respectively [72–75]. However, due to limited evidence in the peri-HCT population, these agents would generally be considered in the second-line setting.

In cases of persistent splenomegaly or otherwise inadequate benefit from initial ruxolitinib therapy, an alternative approved JAKi or clinical trial may be considered [21, 46, 76, 77]. However, clinical data supporting the use of alternative JAKi’s leading up to HCT are limited. Additional options for splenic management pre-HCT include splenectomy [22, 46, 78–81] and splenic irradiation [54, 82–84]; however, neither splenectomy nor splenic irradiation prior to HCT has shown an OS benefit [47, 66, 83] and as such are not consistently performed [85]. In addition, splenectomy is associated with significant morbidity, mortality, and increased risk of complications post-HCT; thus, it is not routinely recommended [78, 86].

In cases where a patient derives benefit from JAKi’s but is not immediately undergoing HCT, we continue JAKi treatment. Patients should be reevaluated for changes in disease status and treatment preferences, with more frequent evaluations for patients with higher-risk disease. However, JAKi’s do not appear to mitigate the risk of leukemic transformation [87], so patients with high molecular risk are recommended to proceed to HCT as soon as possible after a response to therapy.

#### Management of MF-related complications and comorbidities

Monitoring of other MF-related conditions and comorbidities (e.g., anemia/transfusion dependence, iron overload, cytopenias, hepatomegaly, cardiovascular disease, hypertension [portal, intracranial, pulmonary], splanchnic venous thrombosis, esophageal and/or gastric varices) [22, 47, 66, 88–91] is essential as these complications can impact HCT-related outcomes and HCT eligibility. While identifying a donor, transfusion-sparing treatments should be considered for patients with persistent anemia or growing transfusion use to avoid iron overload; supportive treatments including erythropoietin-stimulating agents, danazol, lenalidomide, and luspatercept should be considered as adjunctive therapy [92]. In transfusion-dependent patients, HCT consideration should be expedited to avoid iron overload, which increases the risk of graft failure or poor graft function [93]. If iron overload does occur, chelation therapy may be considered to reduce risk of post-HCT complications [93]. Splanchnic vein thrombosis is another MF-related condition that can occur in ~ 7% of patients with MF; although it is not a contraindication for HCT, it is associated with a higher risk of portal hypertension that can lead to hepatotoxicity, which is itself associated with poorer HCT outcomes [47, 68]. Interestingly, pre-HCT body mass index has been shown to potentially have a small effect on relapse (*P* = 0.031) but no impact on nonrelapse mortality, OS, grade II–IV acute GVHD, or chronic GVHD (all *P* > 0.05), indicating that overweight and obese patients are eligible for HCT [94].

#### Management of accelerated phase MF

Patients who progress to accelerated phase (blasts 10–19%) are at high risk of disease progression. Patients with either advanced phase or blast phase (blasts ≥ 20%) have substantially worse OS [95]. Peri-HCT treatment for these patients should focus on blast percentage reduction with hypomethylating agents (HMA) including azacitidine or decitabine, which can be combined with ruxolitinib for concurrent treatment of splenomegaly and MF-related symptoms [21, 68]. In select cases, more intense induction therapy can be considered, as well as other combinations with HMA, including venetoclax and isocitrate inhibitors [96], although the optimal approach to these patients is unknown. However, durable responses to these treatments are rare. In our practices, we proactively seek HCT as early as possible.

## Clinical case– transplant care and post-HCT considerations

Due to a lack of related donors, an unrelated donor search was conducted, and multiple 10/10 human leukocyte antigen (HLA) matched donors were identified. Based on the patient’s age and comorbidities (HCT Comorbidity Index score of 2, diabetes, and history of arrhythmias), RIC was chosen as the conditioning regimen, with a calcineurin inhibitor and methotrexate for GVHD prophylaxis. A peripheral blood stem cell source was recommended due to faster time to engraftment. Splenic irradiation was performed to optimize the spleen for HCT, and ruxolitinib was tapered to 5 mg bid by the initiation of conditioning.

The patient underwent HCT, and he received ruxolitinib for 1 year post-HCT. He developed grade 2 cutaneous GVHD and grade 1 elevation in his liver function test and was treated with prednisone and ongoing ruxolitinib. The patient was eventually tapered off ruxolitinib and remains in remission of MF.

### Optimization of HCT to improve patient outcomes

#### Timing considerations for HCT

Decisions on HCT timing aim to achieve a balance between risk of transplant-related morbidity and mortality versus the risk of advanced-stage disease or disease transformation if transplant is delayed [22, 79]. The repercussions of this decision were highlighted in a recent large, multicenter, retrospective study that evaluated OS in patients with MF undergoing HCT versus those receiving non-HCT interventions [97]. Regardless of DIPSS risk category, patients undergoing HCT had reduced OS during the first year following transplant compared with the non-HCT control population. However, after 1 year, those with DIPSS categories of intermediate-1 (Int-1) or higher at the time of transplant showed significantly improved OS compared with those who received non-HCT interventions (non-HCT vs. HCT, DIPSS Int-1: hazard ratio [HR], 0.26, *P* < 0.001; DIPSS Int-2 and higher: HR, 0.39, *P* < 0.0001). Given the complexity and inherent risk associated with the decision, patient preference should be a key driver in whether to proceed to HCT early versus treating symptoms and cytopenias alone. In addition, continuous evaluation of risk status, response to JAKi’s, and donor quality and availability are critical in determining when to proceed to HCT if initially deferred [97].

#### Donor considerations

Appropriate donor selection and stem cell source are also important predictors of successful HCT outcomes. Eligible HLA-matched (sibling or unrelated) donor HCT is our preferred choice, which provides better outcomes compared with mismatched unrelated donor HCT [46, 47, 66, 79, 98]. We prefer to use peripheral blood stem cells for HCT over bone marrow, as there is limited supportive evidence for using bone marrow in HCT for MF [46, 79], and retrospective registry-based data suggest lower engraftment rates in bone marrow recipients compared with peripheral blood stem cells [99]. Haploidentical donor transplantation with post-HCT cyclophosphamide-based GVHD prophylaxis can be considered in patients with MF who do not have a suitable matched donor. A recent study based on Center for International Blood and Marrow Transplant Research registry data evaluated 1057 adult patients who received their first peripheral blood HCT between 2013 and 2019 for chronic phase MF. The study showed that matched sibling donors were the preferred donor source in patients with MF, but that post-HCT cyclophosphamide-based platforms with haploidentical donors provided comparable results to unrelated donors for those who did not have an available matched sibling donor, with no differences seen in nonrelapse mortality, relapse, or disease-free survival [100]. Similarly, data from the European Society for Blood and Marrow Transplantation registry indicated that patients who underwent HCT from matched sibling and matched unrelated donors had the lowest nonrelapse mortality and highest OS rates, and that outcomes for mismatched unrelated and haploidentical donor transplants were comparable to one another when patients were treated with cyclophosphamide [101]. However, further prospective studies are needed to validate initial evidence [22, 102].

#### Conditioning regimen intensity

When considering MAC or RIC regimens [20, 54–56], our preferred approach is an RIC regimen with fludarabine plus melphalan, although fludarabine plus busulfan is another common choice, with limited data suggesting that it may be the superior option [103, 104]. RIC regimens have similar OS outcomes compared with MAC conditioning regimens but offer improved outcomes over MAC regarding engraftment and incidence of acute GVHD and are associated with lower nonrelapse mortality [105].

#### GVHD prophylaxis

We recommend calcineurin inhibitors and methotrexate [22, 66, 106] with a consideration to continue ruxolitinib for GVHD prophylaxis in patients who undergo HCT using an HLA-matched donor based on 2 recent phase 2 studies [64, 107]. A prospective, multicenter phase 2 study evaluated the use of ruxolitinib in the pre-, peri-, and 1-year post-HCT setting in patients with MF, with ruxolitinib initiated at a dose of 5 mg bid and increased to 10 mg bid at post-HCT Day 30. Of 43 HCT patients, only 2.4% experienced grade III–IV acute GVHD within 6 months, and 11% had moderate/severe chronic GVHD within 1 year [64]. The study also reported positive 1-year rates for OS (86%), progression-free survival (PFS; 79%), GVHD-free and relapse-free survival (GRFS; 74%), nonrelapse mortality (10%), and disease relapse (10%) [64]. Another prospective, multicenter phase 2 study evaluated the use of ruxolitinib 10 mg bid beginning between 30 and 100 days post-HCT in 63 patients with acute myeloid leukemia or myelodysplastic syndrome [107]. The 6-month cumulative incidence of grade III–IV acute GVHD was 5%, and at 12 months, the incidence of chronic GVHD was 27%, although only 8% overall had chronic GVHD that required systemic treatment [107]. The study also reported favorable survival results, including a 12-month GRFS rate of 70% and 18-month OS and PFS rates of 78% and 68%, respectively [107]. Taken together, results from these 2 studies of ruxolitinib in the pre-, peri-, and post-HCT periods support continuation of ruxolitinib treatment in these patients.

The use of post-HCT cyclophosphamide has expanded over the last decade [103, 108] and has been shown to improve survival rates while reducing the risk of acute and chronic GVHD [109, 110]; however, data are limited outside the haploidentical donor setting in MF. In our practices, we use cyclophosphamide for patients receiving haploidentical donor transplant and those receiving an unrelated donor transplant with less than 7/8 or 9/10 match. However, the potential benefit of cyclophosphamide use needs to be confirmed in larger prospective studies and should be balanced against concerns about graft failure and known adverse effects, including delays in engraftment and immune reconstitution, increased infection risk, hemorrhagic cystitis, and cardiac complications [111–114]. Ex vivo CD34 + selection with a MAC regimen in the absence of additional immune suppressive medication can also reduce GVHD risk [115] and lead to high survival rates [116].

### Post-HCT optimization

Post-HCT management includes close monitoring for graft function/failure, hematologic recovery, GVHD, and disease relapse [22, 46, 117]. In our experience, poor graft function and slow hematopoietic recovery are the main issues that can differentiate MF care post-HCT from other diseases for which HCT is performed. These outcomes are affected by various factors, including degree of splenomegaly, fibrosis grade, thrombocytopenia, choice of conditioning regimen and subsequent patient outcome, donor selection, and stem cell source [22, 79].

#### Graft-versus-host disease

Acute and chronic GVHD remain among the most prevalent complications post-HCT [106]. Rigorous monitoring and prompt treatment of GVHD are the standard of care in patients undergoing HCT for MF [118]. Similar to the integral role discussed earlier of ruxolitinib in patients with MF undergoing HCT, ruxolitinib is approved for treatment of patients 12 years or older with steroid-refractory acute and chronic GVHD in the United States, Europe, and Canada [12, 119, 120] and should be considered standard of care in this setting [121–123]. These indications are based on results from the phase 3 REACH2 and REACH3 trials. In REACH2, patients with steroid-refractory acute GVHD who received ruxolitinib achieved a significantly higher Day 28 overall response rate compared with those in the control group, who received best available care (62% vs. 39%; *P* < 0.001) [121]. Similarly, ruxolitinib treatment for patients with steroid-refractory chronic GVHD in REACH3 resulted in a significantly better overall response rate (50%) at Week 24 compared with best available care (26%; *P* < 0.001) [122]. Based on these studies, the recommended starting doses are 5 mg bid for acute GVHD and 10 mg bid for chronic GVHD [12, 119, 120].

#### Relapse monitoring

Transplant recipients remain at risk of MF relapse [22, 79], occurring in up to 25% of patients [66, 124, 125], although patients with MF can have persistent fibrosis and/or cytopenia after HCT [126, 127], which complicates identification of MF relapse depending on the precise definition used. Importantly, relapse can occur years after HCT, with a cumulative incidence of 14% at a median follow-up of 7 years [128]. Implementing additional approaches to reduce the risk of MF relapse is vital, but the lack of prophylactic therapies to prevent relapse highlights an unmet need for patients that warrants further clinical investigation. Minimal residual disease monitoring using driver mutations as residual disease markers can help identify potential relapse [46, 79]; however, optimal timing of monitoring and sensitivity of testing vary widely across centers [129]. For patients exhibiting MF-related symptoms or splenomegaly after relapse, we consider reintroduction of ruxolitinib, especially for patients with molecularly detected relapse and splenomegaly or constitutional symptoms [130]. In addition to standard therapies for MF, in patients with preemptive molecular relapse or overt clinical relapse, donor lymphocyte infusion may be considered to induce remissions and prevent further disease progression, as recommended by EBMT/ELN [68, 131, 132].

## Conclusions

HCT remains the only curative treatment option for patients with MF. For fit patients with higher-risk disease features, we recommend proceeding to HCT as soon as is feasible to avoid disease progression and complications of cytopenias, in particular, iron overload from transfusions. Ruxolitinib is standard of care in patients with MF and remains an integral part of treatment throughout the HCT process to maintain control of MF disease and symptoms, potentially minimize risk of and treat GVHD, and reduce risk of relapse. Ruxolitinib has multiple retrospective and prospective studies providing safety and efficacy data in the MF HCT setting. In contrast, these data are scarce for other JAKi’s, making them better positioned for second-line or later treatment after ruxolitinib in the HCT setting. There continues to be great interest in the clinical development of new treatments and combination regimens for patients with MF, while HCT approaches continue to improve in parallel. We look forward to the evolving position of HCT in MF treatment in the coming years and expect HCT to remain an essential component of MF management as transplantation practices and patient management continue to improve.

## Data Availability

No datasets were generated or analyzed during the current study.
